# Expressive language development in adolescents with Down syndrome and fragile X syndrome: change over time and the role of family-related factors

**DOI:** 10.1186/s11689-020-09320-7

**Published:** 2020-06-27

**Authors:** Laura del Hoyo Soriano, Angela John Thurman, Danielle Harvey, Sara T. Kover, Leonard Abbeduto

**Affiliations:** 1 The MIND Institute, University of California, Davis, 2825 50th Street, Rm 2101, Sacramento, CA 95817 USA; 2 Department of Psychiatry and Behavioral Sciences, University of California, Davis, Sacramento, USA; 3 Division of Biostatistics, Department of Public Health Sciences, University of California, Davis, Davis, CA USA; 4 Department of Speech and Hearing Sciences, University of Washington, Seattle, WA USA

**Keywords:** Down syndrome, Fragile X syndrome, Longitudinal, Expressive language development, Family-related factors, Adolescence, Conversation, Narration

## Abstract

**Background:**

It is well known that individuals with Down syndrome (DS) or fragile X syndrome (FXS) demonstrate expressive language difficulties beginning early in childhood. It is less clear, however, whether expressive language skills change during the adolescent period in these individuals, and if any of these changes are syndrome specific. Studying this, as well as the role of maternal and family-related factors in expressive language development, may provide the foundation for efficacious interventions for adolescents with DS or FXS.

**Methods:**

In this study, we examined expressive language trajectories, assessed through conversation and narration, in 57 adolescent males with intellectual disability (ID) (20 DS and 37 FXS) in relation to the diagnostic group (DS vs. FXS) and family-related factors (maternal IQ, maternal psychological distress, closeness in the mother–child relationship, family income, and maternal and paternal education) after adjusting for chronological age (CA) and nonverbal cognition.

**Results:**

Changes over repeated annual assessments for males with DS or FXS were observed only during conversation, such as an increase in talkativeness, but a decrease in syntax complexity and lexical diversity. We found a diagnosis-related effect in the change over time in conversational talkativeness favoring those with FXS. Finally, a closer mother–child relationship predicted less decrease over time in lexical diversity during conversation, and participants of mothers who graduated college showed a greater increase in conversational talkativeness over time compared to those of mothers with a high school education.

**Conclusions:**

Our results suggest that, during the adolescent period for males with DS or FXS, there is an increase in the amount of talk produced in conversational contexts, but also a decrease in the quality of the language produced. In addition, our results indicate syndrome-specificity for aspects of expressive language development and reinforce the protective role of family-related factors.

## Introduction

Down syndrome (DS) and fragile X syndrome (FXS) are the two most common genetic causes of intellectual disability (ID). DS is typically caused by an extra copy of all or part of chromosome 21 and has a prevalence of 1 in 1000 to 1 in 1100 live births [1]. FXS results from the expansion of a repetitive sequence of trinucleotides in the *FMR1* gene on the long arm of the X chromosome [2]. Because FXS is an inherited X-linked condition, it affects more males than females, with an incidence of 1.4 per 10,000 males and 0.9 per 10,000 females [3]. Females with FXS are also, as a group, less severely affected than males, with more than 90% of males meeting criteria for ID compared to nearly 33% of females [4, 5]. In this study, we focused on understanding the expressive language development of males with ID associated with DS or FXS, with an emphasis on both common and unique developmental patterns and mechanisms.

Language development is impaired in virtually all individuals with ID [6]. At the same time, however, there is evidence of differences in the profile of language impairments across different etiological conditions, including DS or FXS. Etiological differences are seen, for example, in the severity of delay or impairment in syntax relative to vocabulary and in the expressive versus receptive modalities [6]. Although these etiology-related profile differences can have important functional consequences for the individual and suggest variations in learning mechanisms and/or inputs, it is important to recognize that there are arguably more commonalities than differences among individuals with ID. Indeed, even in ID conditions associated with considerable heterogeneity [7], there are seldom instances of age-appropriate levels of skill in any area of language, which is not surprising given the strong association between language and cognitive development.

Although individuals with DS and those with FXS both show impairments in receptive language, these impairments tend to be consistent with expectations based on their levels of cognitive functioning [8–11]. In contrast, expressive language development typically lags behind receptive language and cognition for both individuals with DS [12] and males with FXS [13]. In addition, several studies have demonstrated that the profile of relative impairment across various areas of expressive language differs between individuals with FXS and those with DS beginning in early childhood [6, 14–16]. However, most of these studies have been based on comparisons between the diagnostic group at a single point in time and often for groups of participants representing wide age ranges [8, 15]. Consequently, little is known about how expressive language profiles change over the course of development for individuals DS or FXS and how they compare. There is a need, therefore, for longitudinal studies in DS and in FXS, with a focus on expressive language being particularly important given the (1) greater impairments in expression than reception and (2) emergence of promising interventions for expressive language in these syndromes [17, 18].

Existing longitudinal studies of expressive language development in DS and in FXS samples have focused largely on early childhood [19, 20]. Adolescence, however, is a particularly important developmental period in individuals with ID due to the fact that there is variation across syndromes in terms of whether intellectual development is stable or slows during this period [21], which can have consequences for language development during this same period. Moreover, expressive language difficulties are known to be a significant predictor of adult independence given the influences of these skills on social success, adaptive functioning, and learning [8].

Existing longitudinal studies also have been limited by their reliance on standardized norm-referenced tests. Although standardized tests are useful to evaluate language performance relative to chronological age (CA) expectations, many of these tests (a) yield only a single or limited number of summary score(s) thereby obscuring potential differences across different areas of language; (b) show limited generalizability to everyday communicative contexts; and (c) are not specifically designed for individuals with ID and thus, often suffer from floor effects when used for this population [22]. As a result, standardized tests may mask variability across areas of expressive language for individuals with ID and mask differences between etiological groups [23].

The current study was designed to address these limitations. We used a longitudinal design to determine the commonalities and differences between individuals with DS and those with FXS in the emergence of their expressive language profiles during the adolescent period. We also controlled for differences in CA and nonverbal cognitive ability. Through the analysis of naturalistic language samples, we focused on characterizing the profiles of relative impairment across different functionally important areas of expressive language. We also examined factors that shape language development in individuals with ID due to DS or FXS. Elucidating the shared and syndrome-specific trajectories of growth across these areas underlying their development will contribute vital information for intervention planning for individuals with DS or FXS. Indeed, several clinical trials of targeted treatments in both individuals with DS and individuals with FXS have utilized gains in expressive language as the primary outcome measure for the treatment [18–21].

### Expressive language development in individuals with DS

All areas of expressive language development are significantly delayed relative to CA expectations in DS [24]. In addition, for individuals with DS, there is considerable research documenting that multiple areas of expressive language lag behind mental age (MA) expectations [15, 25, 26]. At the same time, severity of impairment varies across different areas of expressive language in DS. For example, individuals with DS score less well on measures of vocabulary and syntax when compared to both younger, typically developing (TD) peers [27–30] and similarly aged peers with other ID-related syndromes at similar cognitive level [28, 31–33]. There is also evidence suggesting that expressive syntax skills are more impaired than are expressive vocabulary skills in individuals with DS [34]. In general, pragmatic language delays are often noted in individuals with DS, particularly relative to expectations based on nonverbal cognition [26, 35–38]. That said, pragmatic strengths are often observed in DS, when compared to individuals with other neurodevelopmental disorders [8, 39, 40]. Sensory and motor impairments, as well as cognitive functioning (e.g., nonverbal MA), have been found to contribute to the expressive language problems of individuals with DS; however, these factors account for a relatively small proportion of the variance in expressive language outcomes [21, 23, 29–34, 36], suggesting a need for research to identify additional factors.

### Expressive language development in individuals with FXS

In males with FXS, there are delays relative to CA expectations in multiple areas of expressive language [24]. However, the profile relative to MA expectations differs across areas of expressive language [41].

The syntactic complexity of expressive language is more limited in males with FXS than would be expected based on MA, and this finding is relatively consistent across multiple methods of assessment [28, 42–44]. Expressive pragmatics also appears to be an area of special weakness in FXS, including frequent off-topic and tangential utterances [44, 45], omission of critical story elements in narratives [42], and verbal perseveration (i.e., excessive repetition) of a word, phrase, or topic [46]. In contrast, expressive vocabulary keeps pace with MA expectations in males with FXS when assessed using standardized methods that require naming pictured objects [19]; however, delays relative to MA have been observed in the number of different words used by verbal individuals with FXS in naturalistic language samples [44, 47]. The expressive language profile associated with FXS appears to correlate with cognitive ability [47], behavior, and mental health symptoms (e.g., anxiety and hyperarousal; 48), and various symptoms of autism spectrum disorder (ASD), which are common among males with FXS [48, 49]. Again, however, the variance within and across areas of expressive language has yet to be explained in this population.

### Syndrome comparison studies of expressive language development

Although both DS and FXS are each associated with expressive language difficulties, the severity and areas of relative strength and challenge appear to differ. There do not appear to be syndrome-related differences in vocabulary, at least as indexed by the number of different words produced [8]. Differences have emerged, however, in grammatical morphology and syntax. For example, Price and colleagues (2008) found that males with FXS produced more complex utterances overall and a longer mean length utterance (MLU)—a gross measure of syntactic complexity—in conversation than did MA-matched peers with DS. Similarly, individuals with FXS perform significantly better in terms of measures of expressive syntactic complexity in narration than do those with DS matched on cognitive level [15, 50]. In terms of pragmatics, individuals with FXS produce more noncontingent discourse, stereotyped utterances, and perseverative language than do cognitively matched individuals with DS [8, 48]. Finally, the occurrence of dysfluencies, including filled pauses and repetitions, which are thought to reflect problems with utterance planning, are more common in individuals with DS compared to those with FXS matched on cognitive level [16, 49].

The few longitudinal studies directly comparing the DS and FXS phenotypes have yielded contradictory findings. For example, a study by Roberts et al. [19] reported differences in the developmental trajectory of expressive vocabulary, using the Expressive Vocabulary Test-2 [51], between preschoolers with FXS or DS. In contrast, in a study by Martin et al. [28], no differences were observed in the rate of change in expressive vocabulary, using the Comprehensive Assessment of Spoken Language [52], between boys with DS and boys with FXS who were producing at least 40 words and producing utterances consisting of two or more words. Methodological differences across these studies, differences in participant CA, and the methods of assessment make it difficult to pinpoint why the findings relating to expressive vocabulary differed across these studies. Such findings highlight the need to use measures that have a wide age and ability range of use and to use multiple measures of the same constructs within a study to begin to understand task and context effects on expressive language.

In the present study, therefore, we used naturalistic procedures for obtaining conversational and narrative language samples that have been developed and standardized for use with verbal individuals with ID [16, 53–55]. These expressive language sampling (ELS) measures have a wide age and ability level range of use, from the early school years through adulthood for TD individuals as well as for those with ID [56]. ELS conversational and narrative procedures pose somewhat different demands on speakers and thus, together provide a more comprehensive characterization of expressive language ability than is possible with any single measure. For example, speakers with FXS or DS both display significantly higher MLUs in narration than in conversation [14, 16, 57], suggesting that narration is particularly well-suited to eliciting the upper bounds of syntactic ability and uncovering individual differences [53]. Narrative may “pull” for syntactic complexity in part because it provides numerous opportunities (relative to conversation) to describe relationships between characters and event sequences, all of which are best achieved using multi-clause constructions [58]. In contrast, conversation may allow for more diverse vocabulary use because it is less constraining of the content of the talk [53]. Therefore, ELS procedures allow computation of several measures of expressive language with strong psychometric properties [59–61] and that have been shown to distinguish individuals with ID from TD individuals, individuals with ID at different levels of cognitive ability, and individuals with ID associated with different etiologies, including DS or FXS.

The specific ELS measures used provide indices of talkativeness, which reflects the social-motivational aspect of pragmatics; dysfluencies such as “um” and “er,” which reflects problems in speech planning and execution; lexical diversity, which reflects functional vocabulary size; and mean length of utterance (MLU) in morphemes, which reflects syntax complexity. The utility of these particular measures in tracking developmental change and discriminating typically and atypically developing individuals has been established [16, 53, 55, 58]. Compared to other expressive language measures derived from the typical norm-referenced standardized tests, these expressive language measures are more likely to be generalizable to real-world activities meaningful for the individual with ID [62]. In addition, these measures all display minimal practice effects and have strong test-retest reliability for individuals with FXS and DS in the age range interest, which is important in longitudinal studies that, by definition, involved repeated administration of the same measures [56, 63]. Finally, intervention studies in individuals with different neurodevelopmental disorders have shown change in various ELS measures in the face of a lack of change in standardized tests [64–66]. Thus, we used naturalistic conversation and narration language sampling techniques in a longitudinal design and computed these diverse measures of expressive language performance in verbal individuals with ID associated with DS and with FXS.

### Sources of variation in expressive language development in individuals with DS or FXS

Not all individuals with ID or even a specific genetic disorder such as DS or FXS evidence precisely the same developmental trajectories or phenotypic outcomes. For example, a recent longitudinal study focused on vocabulary acquisition in preschoolers with DS showed that the lowest scoring child at 36 months was nonverbal (i.e., produced zero words), whereas the highest scoring child at the same age produced 243 words [67]. Moreover, when the children were re-assessed 6 months later in this same study, the nonverbal child remained nonverbal, whereas the one with the most developed language had doubled his word production. In a study of infant and toddler males with FXS [68], the lowest scoring child at 18 months was nonverbal, whereas the highest scoring child at the same age was producing ten words. After 6 months, the nonverbal child remained nonverbal, whereas the other child had tripled his production. Understanding the factors accounting for such variation, especially those factors that are modifiable, is critical for intervention efforts.

Identifying the sources of variation in phenotypic development requires a focus on factors at different levels of explanation—genetic, cellular, neural, cognitive, behavioral, and environmental [69]. In this respect, the language environment in which an individual develops can have strong influences on language development [70, 71]. In addition, higher levels of parental education are associated with better expressive language skills in TD children [72, 73], as well as in individuals with ID [6, 74–77]. Studies focused on individuals with FXS or DS have also suggested a positive association of maternal education with expressive vocabulary outcomes after controlling for CA [78]. Not all of these studies, however, have controlled for differences in the children’s cognitive level, which makes it difficult to interpret their findings because variability in children’s language development is likely to be related to cognitive differences [8]. In fact, neither Chapman et al. (2000) nor Estigarribia et al. (2012) found that maternal education predicted expressive syntax in DS after controlling for cognitive level and CA. The language environment, however, may have a variable impact on language development depending on the particular characteristics of the child, such as CA and level of intellectual functioning, as well the specific area of expressive language [79, 80].

Maternal cognitive ability could also impact language development. In the TD population, maternal IQ is related to child cognitive and language development [81, 82]. Although this association has often been attributed to genetics, it is very difficult to isolate the genetic from the environmental factors modulated by maternal IQ, as well as potential epigenetic effects [83], including in genetic syndromes associated with ID [84]. The link between maternal IQ and expressive language development has yet to be adequately investigated for ID; for example, there are reports of a link between maternal IQ and child IQ in FXS [76], but not in DS [85].

Maternal mental health has also been found to be associated with expressive language development in TD children [86] and in individuals with ID [75–77]. Depression, for example, can impede a person’s ability to respond optimally in interactions with their children, which, in turn, can impede language development [87]. Maternal mental health is likely to be a source of within- and between-syndrome differences in DS and in FXS. In particular, the biological mothers of individuals with FXS are at elevated risk for mental health concerns (i.e., anxiety and depression-like symptoms) relative to mothers of children who have DS [88–90]. This is in part because of the former’s own genetic status as carriers of either the *FMR1* full mutation or premutation [91, 92]. At the same time, parents of children with DS tend to report less child-related stress and are more optimistic about their children’s potential outcomes than are parents of children with other IDs [89, 93], which may well shape interactions with their children.

At a more macro-level, there is evidence that socioeconomic circumstances influence language development. For a variety of reasons, children in lower income households tend to receive a significantly less rich language experience [94–96]. At the same time, socioeconomic factors are likely to interact with other dimensions of variability, such as the CA of the child [97, 98] and the child’s phenotype [99–101]. In addition, it is important to note that when more expansive definitions of the verbal environment are applied, the link between socioeconomic environment and language development is not as clear [102].

In summary, multiple facets of the environment influence language development, but with different effects across different areas of language development. Few studies of DS or and FXS, however, have taken a multidimensional approach to investigating family-related influences of language development. Thus, in this study, we examined the relationship between different areas of expressive language development and different family-related factors. We focused on those factors known to be important for TD children and likely to be variable among families with ID: closeness in the mother–child relationship, maternal psychological distress, maternal level of education, maternal IQ, and family income. We were interested in whether and how these factors contributed to language development over and above the effects of diagnosis. These data will be valuable in guiding intervention efforts because although only few of the included family-related factors are to some degree modifiable (e.g., closeness in the mother–child relationship, maternal psychological distress) and thus, potential treatment targets, each of them still needs to be considered to address the special needs of a family (e.g., maternal and paternal level of education, family income).

### The current study

The present study was designed to assess patterns and predictors of change in expressive language measures sampled annually in both conversation and narration across four time points during the transition from late childhood into adolescence in males with ID resulting from either DS or FXS. We took a multidimensional approach, selecting measures that index talkativeness, problems in language planning and execution, size of vocabulary, and syntactic complexity. In terms of predictors, we were especially interested in the roles of family-related factors, but we also considered level of nonverbal cognitive functioning and CA. The following research questions were addressed:

1. Does expressive language change over time in adolescent males with ID associated with FXS or DS? In addressing this question, we were interested in understanding which of the expressive language measures exhibited change over the four visits after accounting for differences in CA and nonlinguistic cognitive ability.
2. In those expressive language metrics that change, do diagnostic group (DS vs. FXS) and family-related factors predict change over time in adolescent males with ID? In addressing this question, we were interested in understanding (a) whether and how change in expressive language differed by diagnostic group after controlling for CA and nonlinguistic cognitive ability and (b) whether and how change in expressive language varied by family-related factors above and beyond CA, nonlinguistic cognitive ability, and diagnostic group.

In these analyses, therefore, we are moving beyond the obvious observation that language is impaired in individuals with ID to asking about the extent to which variations in language are attributable to etiology and/or to family-related factors.

## Method

### Procedures

Families were recruited for this research through newspaper advertisements, nationwide radio announcements, and a university registry of families with children who have developmental disabilities, as well as through postings on internet sites, listservs, and newsletters of developmental disability organizations. Prior to being enrolled in the study, parents of all participants signed informed consent forms approved by the associated Institutional Review Boards. Participants were administered annual assessments of nonverbal (NV) cognition, narrative language and conversational language, across 3 years for a total of up to four assessments. At the initial visit, mothers were administered an IQ assessment along with questionnaires focused on their mental health and the quality of the relationship with their children (described in “Family-related predictors” section). In general, the same examiner administered all assessments to any given participant at every annual assessment. Additionally, the scoring of all test protocols was checked by two examiners and all data entry was double-checked by two research assistants (further details are described in “Predictors” section).

The participants and measures reported on this project are a subset of those previously collected from a larger study (R01HD024356). Eligibility criteria for the larger study required participants to meet the following requirements based on parent report: (a) used speech as the primary mode of communication, (b) regularly used three-word or longer phrases, (c) were native English speakers, and (d) had no major uncorrected physical or sensory impairments that would interfere with performance in the study. Eligibility criteria also required hearing to be directly assessed into establish that pure tone thresholds no worse than 30 dB in the better ear. Several publications have emerged from the project (e.g., [8, 26, 59, 103, 104]), and some have included the conversational and narrative language measures and participants included in the present study; however, no other reports have focused on the longitudinal data from the measures of language included in the present study or the full range of predictors of language examined here.

### Participants

A total of 57 individuals with ID participated: 20 males with DS (CA at first visit: *M* = 12.8, *SD* = 1.9, range = 10.1–15.9 years) and 37 males with FXS (CA at first visit *M* = 12.9, *SD* = 1.7, range = 10.2–16.0 years), along with their biological mothers. See Table 1 for descriptive statistics on family-related measures and Table 2 for descriptive statistics on CA, NV cognition, and expressive language measures of participants at every time point.

**Table 1:** Characteristics of biological mothers and families of individuals with DS or FXS at baseline

Variables	Fragile X syndrome (*n* = 37)	Down syndrome (*n* = 20)	*p* value
Maternal education %			0.14
- Graduated high school - Graduated college - Advanced degree	50.0 30.6 19.4	40.0 55.0 5.0	
Paternal education, %			0.59
- Graduated high school - Graduated college - Advanced degree	51.6 25.8 22.6	60.0 30.0 10.0	
Family Income	$80,000 ($37,000)	$88,000 ($32,000)	0.44
Maternal current CA	41.6 (6.0)	44.2 (6.2)	0.12
Maternal IQ	107.2 (12.1)	110.0 (9.6)	0.38
Maternal GSI (SCL90-R)	54.4 (11.0)	48.2 (9.3)	0.04
Positive Affect Index	25.0 (3.5)	25.3 (2.3)	0.72

*Living with both parents* is missing for one FXS participant; *maternal education* is missing for one FXS participant, *paternal education* is missing for six FXS; *Family Income* is based on interval ratings as stated in the methods section. *Family income* is missing for one DS participant and two FXS participants; *Maternal GSI* missing for one DS participant and two FXS participants; *Positive Affect Index* is missing for one DS participant

*IQ* intellectual quotient, *GSI* general severity index, *SCL90*-*R* Symptom Checklist-90 Revised

Values represent means and standard deviations (in brackets) unless otherwise indicated. Variables followed with a % represent percentages

**Table 2:** Descriptive summaries by diagnosis and time points (DS vs. FXS)

Variables	Fragile X syndrome				Down syndrome			
	Baseline	Time 2	Time 3	Time 4	Baseline	Time 2	Time 3	Time 4
Chronological age	***n*** = **37** 12.9 (1.7) 10.2–16	***n*= 33** 13.7 (1.7) 11.2–17	***n*= 32** 14.9 (1.8) 12.2–18	***n*= 32** 15.8 (1.7) 13.2–19	***n*= 20** 12.8 (1.9) 10.2–15.9	***n*= 18** 13.9 (2) 11.2–17	***n*= 16** 14.8 (2) 12.2–17.9	***n*= 13** 15.4 (1.9) 13.3–19
NV cognition (Leiter-R GS)	***n*= 35** 466.3 (9.4) 446–489	***n*= 30** 468.5 (10) 440–489	***n*= 31** 468 (9.5) 446–489	***n*= 30** 468.5 (10.2) 438–489	***n*= 19** 460.3 (7.2) 442–474	***n*= 17** 463.3 (8.4) 450–480	***n*= 15** 465.3 (9.1) 449–483	***n*= 13** 469.5 (9.8) 452–490
NV cognition (Leiter-R age equivalents)	***n*= 36** 5.4 (1.1) 3.3–8.3	***n*= 33** 5.5 (1.3) 2.8–9.5	***n*= 32** 5.5 (1.1) 3.4–8.2	***n*= 32** *5.5* (*1.2*) *2.9-8.6*	***n*= 20** *4.7* (*0.7*) *3.1–6.4*	***n*= 18** *4.9* (*1.1*) *2.7–7.4*	***n*= 16** *5.2* (*1*) *3.6–7.5*	***n*= 13** *5.7* (*1.1*) *3.8–8*
Conversation	***n*= 33**	***n*= 33**	***n*= 28**	***n*= 30**	***n*= 17**	***n*= 16**	***n*= 11**	***n*= 10**
Syntactic complexity	4.0 (1.4) 1.9–7.0	3.9 (1.7) 1.3–8.4	3.8 (1.6) 1.2–7.2	3.3 (1.5) 1.2–7.4	2.9 (0.8) 1.8–4.3	2.9 (0.6) 2.0–4.1	3.0 (1.1) 1.4–5.1	2.8 (1.2) 1.6–5.5
Lexical diversity	86.9 (28.2) 19–138	83.8 (33.0) 17–141	82.5 (31.4) 21–136	73.7 (30.9) 24–135	64.9 (15.8) 40–90	65.6 (17.5) 39–101	65.4 (23.3) 27–103	63.3 (16.9) 43–86
Talkativeness	12.7 (3.5) 6.5–20.2	12.9 (4.1) 4.5–20.4	14.8 (3.4) 6.0–22.5	15.1 (5.0) 3.6–23.9	12.9 (3.6) 6.9–17.9	12.8 (4.3) 3.3–19.2	11.4 (3.1) 6.7–16.8	11.8 (4.1) 5.4–18.9
Dysfluency	0.2 (0.1) 0–0.5	0.2 (0.1) 0–0.5	0.1 (0.1) 0.02–0.5	0.2 (0.1) 0.01–0.4	0.2 (0.2) 0.01–0.7	0.3 (0.2) 0.03–0.6	0.2 (0.1) 0.04–0.5	0.2 (0.2) 0.02–0.6
Narration:	***n*= 34**	***n*= 31**	***n*= 31**	***n*= 29**	***n*= 18**	***n*= 15**	***n*= 12**	***n*= 10**
Syntactic complexity	4.7 (1.7) 1–8	4.7 (1.7) 1.5–8.7	4.3 (1.8) 1.5–8.5	4.4 (1.7) 1.6–8.5	3.5 (1.4) 1.2–6	3.7 (1.2) 2–6.2	4.6 (1.5) 1.6–6.8	4.1 (2.2) 1.9–8.2
Lexical diversity	61.0 (28.7) 1–128	62.1 (28.6) 11–123	61.1 (33.0) 15–122	55.1 (27.5) 9–108	40.8 (23.6) 5–79	43.7 (25.2) 2–83	57.3 (26.4) 24–103	44.1 (25.8) 15–87
Talkativeness	11.4 (4.7) 0.3–22.1	11.3 (4.4) 4.1–17.4	12.1 (4.9) 4.1–22.5	12.1 (5.6) 3.9–29.9	7.0 (3.0) 3.2–14.1	6.4 (3.1) 0.3–12.2	8.0 (3.3) 3.5 (13.1)	5.9 (1.7) 3.2–8.1
Dysfluency	0.2 (0.1) 0–0.3	0.1 (0.1) 0–0.5	0.1 (0.1) 0–0.3	0.1 (0.1) 0–0.5	0.3 (0.2) 0–0.7	0.3 (0.2) 0.02–0.8	0.2 (0.2) 0.01–0.7	0.3 (0.2) 0.04–0.7

Descriptive summaries of CA, NV cognition, and age equivalent (Leiter) and expressive language measures derived from conversation and narration (ELS) are represented by diagnosis and time point. Values represent sample size (bold/italic font), mean followed by standard deviation (in brackets) and range for each measure. Note that individuals may miss a visit, but return for a later visit; for example, in DS, 15 of 17 with conversation samples at time 1 were seen at time 3 or time 4 as were 14 of 18 with narration samples, so although sample sizes at the later time points are much lower than at time 1, most individuals are still contributing to at least one of these later time points

*CA* chronological age, *NV* nonverbal, *GS* growth score

### Measures

#### Dependent measures

##### Expressive language

Expressive language skills were assessed through both a conversation and a narration activity, each designed to elicit spontaneous language in meaningful social activities [53]. These activities were scripted to increase consistency in examiner behavior, content of talk, and nature of the interaction, thereby ensuring comparability of the language samples across participants and occasions of measurement [22, 56].

In conversation, each participant took part in an interview-style conversation with an examiner. The main goal of the examiner was to elicit as much talk from the participant as possible for 12 min. The activity was introduced by saying that the examiner and participant would sit and talk for about 10 min to get to know each other a little better. Then the examiner moved to an idiosyncratic opening topic elicited from a previous interview with the caregiver (e.g., “I was talking to your mother and she told me that you love origami, that sounds very interesting to me. Tell me about that”). Then after, no more than 3 min on the idiosyncratic topic, the examiner moves to the first topic on a list (e.g., school day). The examiner attempts to use mainly open-ended prompts (e.g., “Tell me everything you did in school yesterday”) and to limit her own speech. Reasonable standardization was ensured by use of a standard set of topics (e.g., school day, after-school, pets, companions, playing games, and vacations) and broad follow-up questions and prompts (e.g., “What do you like about [topic]? Tell me all about [topic].”). The examiner attempted to introduce at least three topics during the 12 min. If a topic did not seem of interest to the participant, the examiner moved to the next topic trying at least one or two follow-up prompts for each topic. If a participant introduced topics not on the list, the examiner would keep that topic going by using appropriate follow-ups. If the examiner exhausted the predetermined topics but did not get 12 min, up to two more idiosyncratic topics were introduced by the examiner. Two versions of the topic list were created. The versions were alternated across participants and time. Approximately half of the participants received version A and half received version B. Topic order within each version was the same across participants.

In narration, participants were shown one of two wordless picture books, *Frog Goes to Dinner* or *Frog on His Own* [105], counterbalanced across participants and time. The examiner introduced the book and showed each page for approximately 10 s, allowing the participant to look through the entire story. The participant was then asked to tell the story to the examiner. This second time through the book, the experimenter controlled the turning of the pages turned a page 5 to 7 s after the participant had finished narrating to ensure that the participant had finished talking. Prompting was limited largely to the first page of the story, with the nature and timing of the prompts standardized.

Conversations and narrations were audio-recorded and later transcribed and analyzed using the Systematic Analysis of Language Transcripts (SALT [106];) software. The following dependent measures were derived from participant’s language samples during conversation and during narration with the unit of analysis being the C-unit (i.e., an independent clause and its modifiers, which could include dependent clauses): (1) *talkativeness* (number of C-units attempted per minute), (2) *dysfluency* (proportion of C-units that contained a verbal dysfluency, false start, or filler), (3) *lexical diversit*y (number of different words in 50 complete and intelligible C-units or the full sample if less than 50 C-units), and (4) *syntactic complexity* (mean length of utterance in morphemes, or MLU, for complete and intelligible C-units). Each of the four dependent measures were included in our statistical models for conversation and for narration, with a total of eight dependent variables. Note that the first 10 min of conversation were transcribed as was the entire telling of the story on the second viewing of the book for narration. See Abbeduto et al. [16] for details of the transcription process. The scripted versions of conversation and narration described here yield highly reliable and valid estimates of talkativeness, dysfluency, lexical diversity, and MLU for individuals with ID of the ages studied and allow for discrimination of different etiological groups [55, 56, 60, 103]. This is true even with samples per participant considerably less than the 100 utterances traditionally thought to be an adequate target for research [30, 44]. In fact, there is some evidence that even samples as brief as 3 min yield data sufficient for discriminating among groups of different language abilities [14, 16, 58].

We randomly selected for independent transcription ~ 8% of the 358 samples collected to estimate inter-transcriber agreement: 19 samples (ten narrations) from participants with DS and 22 samples (12 narrations) from participants with FXS. Dimensions of transcription relevant to the dependent measures of this study were assessed. For some metrics, agreement was a bit higher for FXS than for DS, perhaps reflecting the latter’s poor intelligibility. Inter-transcriber agreement averaged 87% (DS) and 83% (FXS) for segmentation into C-units, 89% (DS) and 89% (FXS) for identification of partly or fully unintelligible C-units, 99% (DS) and 97% (FXS) for identification of complete C-units, 93% (DS) and 96% (FXS) for identification of C-units containing mazes, 74% (DS) and 79% for identification of the exact number of morphemes in each C-unit, 74% (DS) and 83% (FXS) for identification of the exact number of words in each C-unit, and 80% (DS) and 81% for the exact lexical and morphemic content of each C-unit. For the last three dimensions, we required that the two transcriptions were in complete agreement for a C-unit, which is a conservative approach to agreement [56].

### Predictors

#### Youth-related predictors

##### Diagnostic group

All participants were required to have been diagnosed with FXS or DS, with molecular confirmation based on DNA analysis for participants with FXS and chromosome analysis to document trisomy 21 or translocation for participants with DS. Diagnostic group (DS vs. FXS) was a predictor in the analyses.

##### Nonverbal cognition

The Brief IQ subtests of the Leiter International Performance Scale-Revised (Leiter-R [107];) were administered. The Leiter is nonverbally administered (i.e., pantomimed) and no verbal responses are required. The subtests of the Brief IQ are: Figure Ground, Form Completion, Sequential Order, and Repeated Patterns, with the former two subtests focused on the visualization domain and the latter two on fluid reasoning. Growth scores (GSs) were used in our longitudinal analyses because they are equal-interval scores, also known as derived rasch scores, provide a measure of absolute ability, and demonstrated a normal distribution in our sample, unlike other type of scores (e.g., IQ scores showed floor effects, especially for the DS group, and age-equivalent scores which represent the median CA at which a raw score was obtained within the norming sample and are not on an equal interval scale [23, 108]). Descriptive statistics regarding mental age equivalents of participants are also provided, however, at every time point for reference (see Table 2).

##### Initial chronological age

Given that the chronological age (CA) of our participating youth at the first visit ranged from 10.1 to 16.0 years, CA at first visit was included as a potential predictor of change in expressive language variables over time.

#### Family-related predictors

##### Maternal IQ

The Kaufman Brief Intelligence Test, Second Edition (KBIT-2 [109];) was administered on an individual basis to mothers. The KBIT-2 is a standardized measure consisting of two verbal subtests that require pointing to pictures representing words spoken by the examiner (Verbal Knowledge) or responding verbally to questions from the examiner (Riddles) and one NV subtest (Matrices) requiring completion of visually depicted puzzles by selecting the best option from among several. We used the composite standard score, which for the norming sample has a *M* = 100 and *SD* = 15.

##### Maternal psychological distress

The Symptom Checklist-90 Revised (SCL90-R [110];) is a 90-item self-report instrument that queries a range of mental health symptoms. Each symptom is rated from 0 (no distress) to 4 (extremely distressed). Scores for nine primary symptom dimensions are derived (e.g., obsessive-compulsive, depression, anxiety). We used the general severity index (GSI) T-scores (*M* = 50, *SD* = 10 for the norming sample) as our index of maternal psychological symptoms of distress.

##### Closeness in the mother–child relationship

The Positive Affect Index (PAI [111];) was used to assess the quality of the mother–child relationship. Five self-report items focused on the mother’s perception of the youth’s reciprocated closeness (i.e., how close she believed the youth felt toward her; Child-PAI: sum of items 1 to 5) were used from this 10-item self-report scale. Items rated understanding, trust, fairness, respect, and affection in the relationship on a 6-point scale. Possible scores for Child-PAI range from 5 to 30, with higher scores reflecting a higher quality of the relationship.

##### Maternal and paternal level of education

Mothers were asked to indicate both their own level of education and that of their son’s other parent, which in our sample was the father—if there was a second parent. Mothers selected the highest level of education achieved using categories ranging from 1, complete elementary-middle school (K-8th) to 5, advanced degree.

##### Family income

Mothers were asked to report family income by selecting a level from < $10,000 to > $150,000, with the levels defined in $10,000 increments.

### Data Analysis

Repeated measures, random effects models were used to assess patterns of change in each expressive language measure (talkativeness, dysfluency, lexical diversity, and syntax complexity) separately for conversation and narration, for a total of eight outcomes, across four annual time points. In those measures that changed over time, we further evaluated whether syndrome or family-related variables were associated with change over time for our sample of individuals with DS or FXS. To better meet the assumptions of the models, dysfluency in narration and conversation and syntax complexity in conversation were transformed with the natural logarithm prior to analysis.

Each analysis included the full sample of participants (participants with DS or FXS). To address the first aim of the study, we evaluated change over time for each dependent measure by including time in study (time in years since the baseline visit; 0 for baseline and 1, 2, or 3 for follow-up visits) as the primary independent variable; models further controlled for CA at baseline (centered at the mean age of 12.9 years), its interaction with study time, and Leiter-R GS at each visit as a time-varying covariate. To address the second aim of the study, we considered factors associated with change by adding a variable (such as diagnosis or a family-related variable) along with its interaction with time to the models for outcomes that exhibited change. We considered each family-related variable (KBIT-2 Composite IQ, PAI, GSI T-score (from SCL90-R), maternal and paternal levels of education, family income) in separate models due to the small sample size, but included diagnosis in each model to determine if the family-related factor was associated with change independent of diagnosis. In these models, the estimated coefficients (βs) for “time” and any interactions with “time” represent estimates of annual change and how other factors (such as syndrome) were associated with that change; coefficients for terms that do not include “time” reflected associations with the baseline level of an outcome (i.e., intercept). In particular, for non-transformed outcomes, the coefficients should be interpreted as the average difference in either the level or rate of change associated with a one-unit difference in the predictor. For the outcomes transformed using the natural log, the exponentiated coefficient may be interpreted as the percentage difference in the level or rate of change associated with a one-unit difference in the predictor.

Random intercepts were included in all models to account for between-person variability in overall starting level. When supported by the data, random slopes were also included to account for between-person variability in change. Robust sandwich estimators were used for standard error estimation due to the small sample sizes. All analyses were conducted in SAS version 9.4, with a *p* value less than 0.05 considered significant.

## Results

### Descriptive statistics

Table 1 provides a descriptive summary of the family characteristics for the participants at baseline. Consistent with previous findings in the literature [89], maternal psychological distress scores were lower for the DS group than for the FXS group, although the means for neither group was in the clinical range. The two groups were not significantly different on any of the other characteristics. Table 2 provides a descriptive summary of participants’ CA, NV cognition, and expressive language sampling scores for each time point. Although there was a decrease in sample size for the later time-points in both groups, there were no significant differences in CA or expressive language sampling scores at baseline between those who dropped-out of the study early and those that remained in the study (*p* > 0.5, except for dysfluency in conversation and syntax complexity and lexical diversity in narration for DS (*p* > 0.1) and talkativeness in conversation for FXS (*p* = 0.1)).

### Does expressive language (syntax complexity, lexical diversity, talkativeness, and dysfluency) in conversation and narration change over time in adolescent males with ID associated with DS or FXS?

**Fig. 1:**
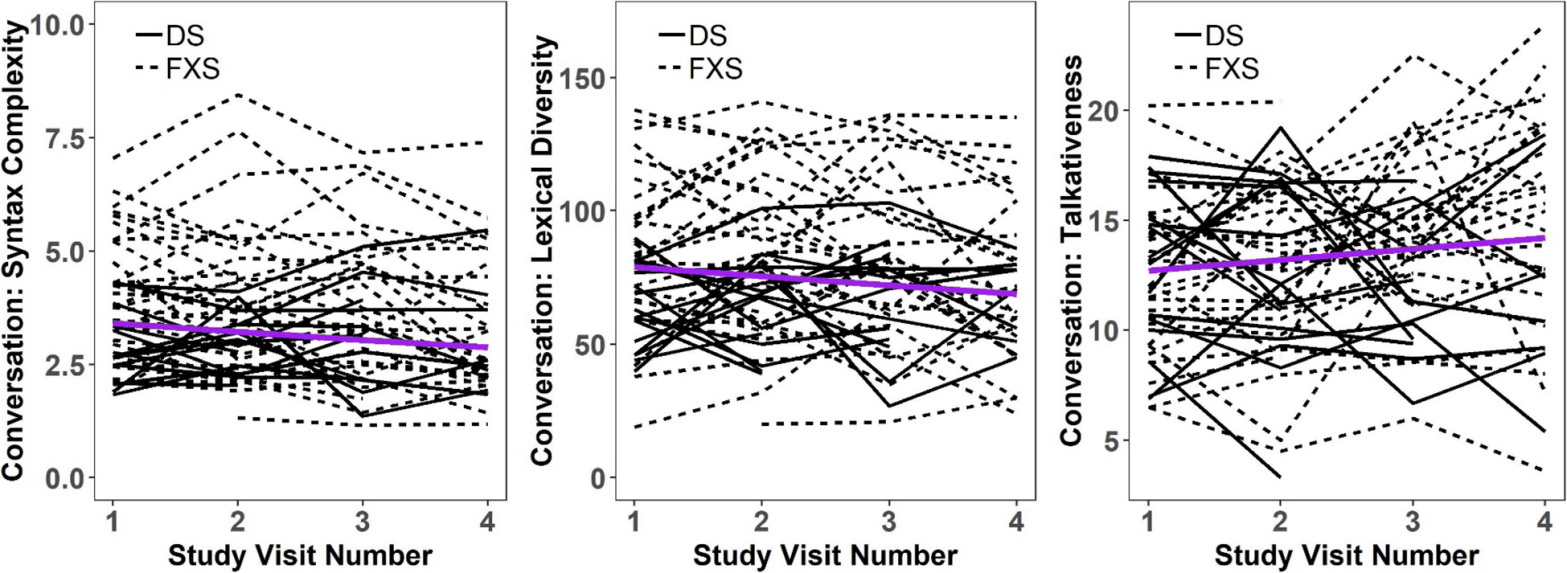
Spaghetti plots representing syntax complexity, lexical diversity and talkativeness for the combined DS-FXS sample. Dashed lines represent participants with FXS; continuous lines represent participants with DS. The purple line represents estimated average trajectories over time for the combined sample (DS + FXS)

In models that included baseline CA and nonverbal cognitive ability (as indexed by Leiter-R GS) at each visit, we estimated the annual rate of change across up to four visits for each expressive language measure in conversation and narration among our adolescent males with ID (Table 3). Only measures during conversation exhibited change in our sample (Fig. 1 and Table 3). Syntax complexity decreased by 6% per year, whereas lexical diversity decreased by 3.4 words per year. However, conversational talkativeness increased by 0.5 units per year (1.5 C-units higher per minute after 3 years).

**Table 3:** Longitudinal change of ELS measures of participants with DS or FXS

Context [β (SE), *p*]	Syntax complexity	Lexical diversity	Talkativeness	Dysfluency
Conversation	− **0**.**06** (**0**.**02**), ***p*** = **0**.**002**	− **3**.**4** (**1**.**4**), ***p*** = **0**.**02**	**0**.**5** (**0**.**2**), ***p*** = **0**.**02**	− 0.007 (0.02), *p* = 0.7
Narration	− 0.07 (0.08), *p* = 0.4	− 1.7 (1.2), *p* = 0.2	0.05 (0.2), *p* = 0.8	− 0.02 (0.02), *p* = 0.5

Dysfluency in narration and conversation and syntax complexity in conversation were transformed with the natural logarithm prior to analysis. The estimated coefficients (*β*s) represented estimates of annual change in a specific measure for the entire sample of participants (DS + FXS). Models included baseline CA, its interaction with time and time-varying Leiter-R GS. Bolded values have *p* < 0.05

*CA* chronological age, *GS* growth score

### Do diagnostic group (DS vs FXS) and family-related factors predict change over time in expressive language in adolescent males with ID?

**Fig. 2:**
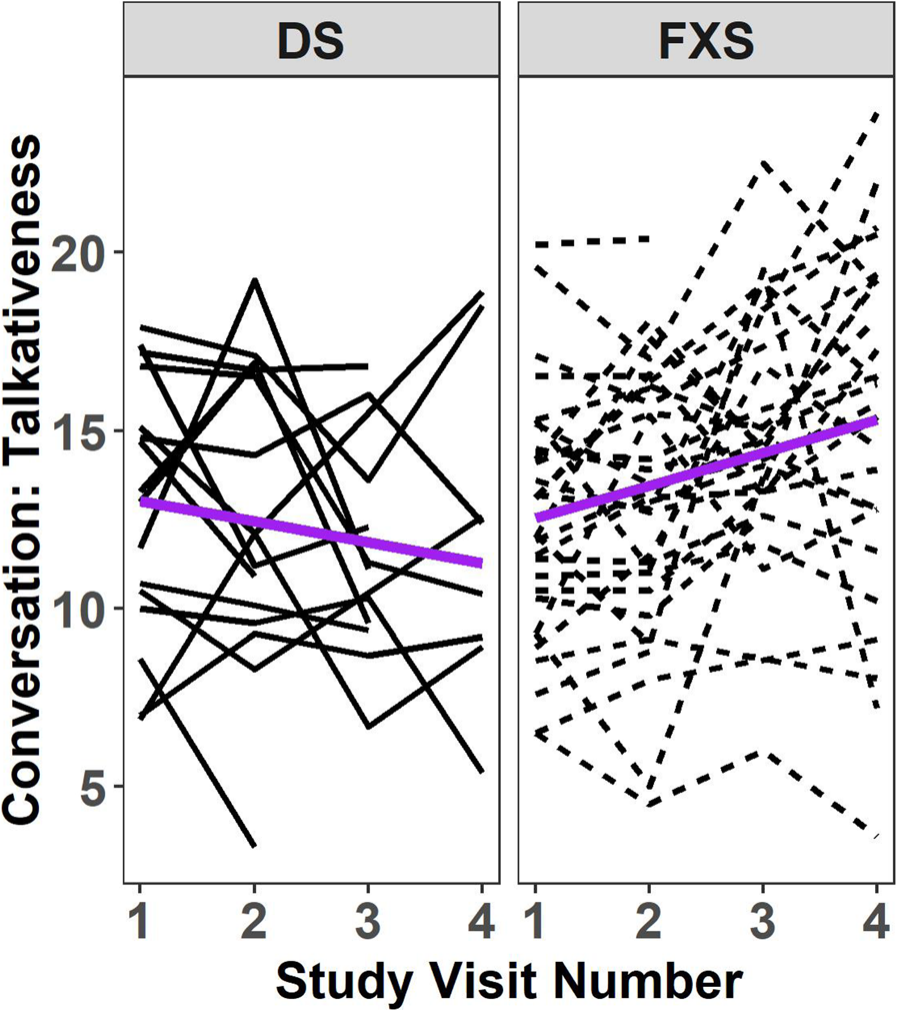
Spaghetti plots representing talkativeness during conversation separately DS and FXS sample. Dashed lines represent participants with FXS; continuous lines represent participants with DS. The purple line represents estimated average trajectories over time for each diagnosis (DS vs. FXS)

After controlling for baseline CA and Leiter-R GS at each visit, diagnostic group (FXS vs. DS) accounted for differences only in the magnitude of change over time in conversational talkativeness (see Fig. 2 and Table 4). In particular, males with DS increased less over time in conversational talkativeness than did males with FXS (β = − 1.5, SE = 0.4, *p* < 0.001, 1.5 fewer C-units per minute per year). There were also differences at baseline between the diagnostic groups in these measures. On average, both syntax complexity and lexical diversity were lower in DS than in FXS; syntax complexity was 21% lower and lexical diversity was 15.9 words lower at baseline.

**Table 4:** Association of diagnostic group with level and change of conversational ELS measures, while controlling for CA and nonverbal cognition

Variable [β(SE), *p*]	Syntax complexity	Lexical diversity	Talkativeness
DS	− **0**.**24** (**0**.**08**), ***p*** = **0.003**	− **15**.**9** (**5**.**3**), ***p*** = **0.004**	0.49 (0.99), *p* = 0.6
Time	− **0.06** (**0**.**02**), ***p*** = **0.003**	− 3.3 (1.7), *p* = 0.06	**0.92** (**0**.**21**), ***p*** < **0.001**
DS x Time	− 0.02 (0.03), *p* = 0.6	− 0.42 (2.18), *p* = 0.8	− **1.5** (**0**.**4**), ***p*** < **0**.**001**

Syntax complexity was transformed with the natural logarithm prior to analysis. The coefficient for DS represents the average difference between DS and FXS at baseline in ELS. The coefficient for time is the estimated annual change in FXS and the coefficient for DS × time is the estimated difference in annual change between DS and FXS. Models include baseline CA, its interaction with time, and Leiter-R GS at each visit. Bolded values have *p* < 0.05

*DS* Down syndrome, *CA* chronological age, *GS* growth score

**Fig. 3:**
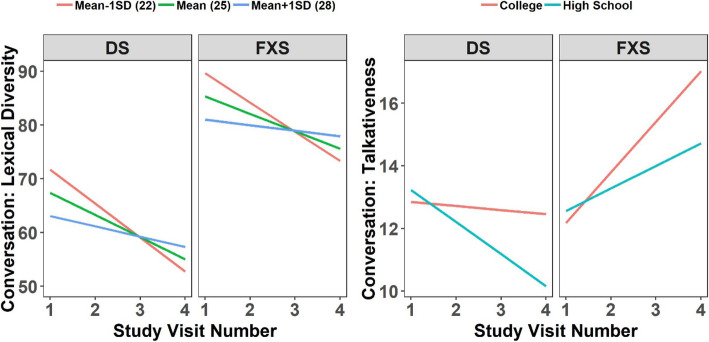
Changes over time in lexical diversity and talkativeness during conversation predicted by family-related variables. Plot **a** represents estimated average trajectories over time in lexical diversity during conversation for individuals with maternal perception of child’s reciprocated closeness at the mean (green), 1 standard deviation (SD) below the mean (red) and 1 SD above the mean (blue). Closer mother-child relationship was positively associated with the rate of change over time. Plot **c** represents estimated average trajectories over time in talkativeness during conversation for individuals with mothers who graduated college (red), and mothers who graduated high school (blue)

We further evaluated whether any of the baseline family-related variables predicted the rate of change in the conversational measures that exhibited change (see Fig. 3 and Table 5). No maternal or family-related variable predicted change over time in syntax complexity in conversation. However, PAI-child score was related to changes over time in lexical diversity in conversation, and maternal education predicted change over time in talkativeness in conversation. In particular, higher levels of the PAI-child variable predicted a lesser decrease in conversational lexical diversity. Finally, for talkativeness in conversation, those participants whose mothers had graduated college increased more over time than those whose mothers had only a high school education.

**Table 5:** Association of family related variables with level and change of conversational ELS measures, while controlling for CA, diagnostic group (DS or FXS) and nonverbal cognition

Variable [β (SE), p]	Syntax complexity	Lexical diversity	Talkativeness
Maternal education			
High school	REF	REF	REF
College	0.1 (0.1), *p* = 0.3	3.3 (7.7), *p* = 0.7	− 0.4 (1.0), *p* = 0.7
Advanced degree	0.004 (0.2), *p* = 0.9	− 1.2 (10.2), *p* = 0.9	0.4 (1.6), *p* = 0.8
Maternal education × time			
High school	REF	REF	REF
College	0.002 (0.03), *p* = 0.9	2.8 (3.2), *p* = 0.4	0.9 (0.3), *p* = 0.009
Advanced degree	0.05 (0.04), *p* = 0.2	4.6 (2.9), *p* = 0.1	− 0.4 (0.5), *p* = 0.4
Paternal education			
High school	REF	REF	REF
College	− 0.2 (0.2), *p* = 0.3	− 10.9 (10.7), *p* = 0.3	− 0.4 (1.1), *p* = 0.7
Advanced degree	0.1 (0.1), *p* = 0.2	6.7 (8.2), *p* = 0.4	− 2.9 (1.2), *p* = 0.02
Paternal education × time			
High school	REF	REF	REF
College	0.08 (0.04), *p* = 0.09	7.6 (3.6), *p* = 0.04	0.2 (0.4), *p* = 0.5
Advanced degree	0.003 (0.04), *p* = 0.9	0.9 (2.8), *p* = 0.7	0.3 (0.5), *p* = 0.5
Maternal IQ	0.004 (0.004), *p* = 0.3	0.1 (0.3), *p* = 0.7	0.05 (0.04), *p* = 0.2
Maternal IQ × time	0.0009 (0.001), *p* = 0.4	0.1 (0.1), *p* = 0.2	− 0.01 (0.02), *p* = 0.4
Maternal GSI	− 0.001 (0.004), *p* = 0.8	0.2 (0.3), *p* = 0.6	0.04 (0.04), *p* = 0.3
Maternal GSI × time	0.0004 (0.001), *p* = 0.8	− 0.1 (0.1), *p* = 0.3	− 0.0003 (0.02), *p* = 0.9
Family income	0.01 (0.01), *p* = 0.2	0.8 (0.8), *p* = 0.3	− 0.1 (0.1), *p* = 0.5
Family income × time	0.003 (0.004), *p* = 0.5	0.5 (0.3), *p* = 0.1	− 0.01 (0.04), *p* = 0.8
PAI	− 0.02 (0.01), *p* = 0.2	− 1.4 (1.1), *p* = 0.2	0.07 (0.1), *p* = 0.6
PAI × time	0.003 (0.004), *p* = 0.4	0.7 (0.3), *p* = 0.03	− 0.02 (0.04), *p* = 0.7

Syntax complexity in conversation was transformed with the natural logarithm prior to analysis. Although paternal education has levels with a *p* value < 0.05, the overall assessment of paternal education with change in lexical diversity (*p* = 0.1) and with baseline level of talkativeness (*p* = 0.07) were not significant. Separate models were fit for each family-related variable. The terms with “Time” quantify the average difference in annual change associated with a 1-unit change (or relative to the reference group) in the predictor. Terms without “Time” correspond to associations with baseline levels (“intercept”). All models included baseline CA, diagnosis, their interactions with time, and time-varying Leiter-R GS

*REF* reference group, *GSI* general severity index, *PAI* positive affect index

## Discussion

This study was designed to identify areas of change in expressive language over time and predictors of change during adolescence in males with DS or FXS. Results from the present investigation suggest that adolescence is a significant period for expressive language development in youth with DS or FXS. In conversation, syntactic complexity, lexical diversity, and talkativeness changed over time. The fact that talkativeness increased with time for the full sample of participants (regardless of diagnostic group) suggested a growing ability or an increase in motivation to engage in linguistic interactions. However, no other increases were observed, and measures of lexical diversity and syntax complexity in conversation actually showed a decline over time for the full sample. It is unlikely, however, that this decline reflects a regression, or loss of lexical and syntactic skills. Instead, decreased lexical diversity and syntactic complexity may relate to task demands. In DS, there is evidence of early declines in concrete expressive language skills (i.e., semantic fluency) related to the early onset of Alzheimer’s disease-related [112]; however, that decline is believed to begin several years post adolescence. Most importantly, no CA-related declines in lexical diversity or syntax complexity assessed in narration were observed for either diagnostic group, suggesting that something about the social or processing demands of conversation that are driving the decline. The declines in conversational performance seen may reflect a tradeoff between quantity and quality; that is, verbal individuals with ID, whether due to DS or FXS, may attempt to participate more fully in linguistic interaction as they grow older and normative expectations to talk increase, but they may be able to do so only by simplifying their contributions. Presumably, such simplifications are the cost of meeting the real-time dynamic requirements of increased participation in linguistic interaction.

We found minimal diagnostic group-related differences in expressive language in the rate of change over time. When adjusting for CA at baseline and level of NV cognitive ability at each visit as a time-varying covariate, diagnostic group-related differences were only observed in the rate of change in conversational talkativeness. Individuals with FXS showed larger increases in this variable compared to those with DS. We hypothesize that the superior lexical and syntactic skills of individuals with FXS at baseline may motivate, and make easier from a processing perspective, participation in conversation compared to individuals with DS. The latter’s more limited lexical and syntactic skills might lead to a reticence to participate. Further investigation, though, is needed to confirm this hypothesis.

Family-related factors also contributed to the rate of change with time in expressive language over and above the contributions of CA, NV cognition, and diagnostic group. In particular, higher levels of maternal education were associated with a greater increase in talkativeness in conversation over time. Moreover, greater closeness in the mother–youth relationship predicted a lesser decline over time in lexical diversity in conversation. These factors are likely to be proxies for a constellation of more proximal variables, including the ways in which parents talk to, and interact with, their children. Understanding the pathways of influence of maternal education and perceived closeness will be important for designing individualized interventions that target parental behavior [77, 78, 113, 114]. At the same time, however, it is important to recognize the bidirectional and dynamic nature of the youth with DS or FXS and the environment provided by parents.

Although our focus in this study was understanding change over time in expressive language, we also found diagnosis-related differences at baseline (i.e., the first annual assessment). First, males with DS scored lower in lexical diversity in conversation at baseline than did males with FXS. This finding contrasts with previous studies that have failed to find differences in lexical diversity between verbal individuals with DS and those with FXS [14, 16]. In part, this may reflect differences in the demands of the tasks used to assess expressive vocabulary. Even with expressive language sampling procedures, task differences have been found with conversation being found to elicit more diverse vocabulary than narration for a variety of typical and atypical populations [14]. Second, we found lower levels of syntax in expressive language samples of individual with DS than those with FXS. This finding is consistent with previous studies in demonstrating an especially severe delay in individuals with DS [12], with development lagging behind CA- and cognitive level-matched individuals with FXS [15, 16, 20, 30]. Not all previous studies, however, have found these diagnosis-related differences in expressive syntax [8, 14, 59, 115]. Also, in contrast to previous studies, the diagnosis-related differences in syntax were seen in conversation but not in narration in the present study. Conversation may place greater social demands on planning turns and with less visual support relative to narration, which may actually hinder syntactic performance more in verbal individuals with DS than in those with FXS.

## Limitations and future directions

In closing, we acknowledge several limitations of this study. First, the sample size was relatively small; therefore, our conclusion should be taken cautiously. As a result, the small sample size required that we examine several predictors separately rather than together, which precluded determining their relative contributions to expressive language outcomes as well as any differences there might be in factors associated with expressive language in FXS and DS. Second, the SCL-90 is a measure of current maternal psychological distress, a variable that could change significantly over time. It also is possible that scores on the PAI could change over time, reflecting shifts in maternal perception of the mother–adolescent relationship. It would be interesting to track change in maternal stress and psychological state. Indeed, our original intent was to follow up with mothers at time 4 regarding these measures. However, we had a high number of mothers who chose not to participate in such assessment; therefore, we decided to only include baseline data in our analyses. Third, we examined only a few possible dimensions of the environment and we did not examine more proximal variables, such as the quality of parent–adolescent interactions. Research in this latter area would have direct implications for intervention. Fourth, females with FXS were excluded from our analyses, as well as females with DS in order to preserve sex-matching when comparing phenotypes. As in most previous studies, we excluded females with FXS because of the relatively lower severity of impairment compared to males. However, it is also true that many females with FXS display expressive language impairments [116] and thus, following them longitudinally would be important in future studies. Fifth, given the high number of individuals with FXS meeting criteria for an ASD diagnosis or displaying ASD-like symptoms [117], and previous literature on FXS showing an effect of autistic behaviors on communication development during childhood [118], ASD symptoms would have been an informative predictor, but were unavailable for participants with DS. Sixth, there was some missing data, although there were no significant differences between those with complete data and those with missing visits, keeping in mind that power to detect differences was limited by sample sizes. For all these reasons, larger studies will help to clarify our understanding of expressive language development and factors associated with it in DS and in FXS.

It is also important to note that given the nature of the current study, participants in the larger study from which data were taken were required to be verbal. This eligibility criteria fosters important bias into the sample, since there is a subgroup of adolescents with DS or FXS who are non-verbal and are not being represented in this study. Therefore, we acknowledge that this study does not address the full phenotypic spectrum for either disorder. Finally, our findings may not generalize to other age periods. For example, the observed changes with age aimed to vary with the nature of the disability might differ dramatically at a later age as a consequence of the high risk of developing the neuropathology associated with Alzheimer’s disease and the resulting dementia-like symptoms for those with DS [119]. In summary, although the current study uses a multidimensional approach to examine patterns and predictors of change in expressive language during the transition from late childhood to adolescence in males with DS or FXS, further longitudinal investigations, including other potential predictors of interest (i.e., father–youth closeness, biomarkers of interest) with larger samples including females are needed to extend our results.

## Conclusion

Collectively, our results suggest that adolescence is an important period in expressive language development for youth with DS or FXS, during which they increase the number of attempts at communication, but also decrease the quality of their speech in terms of syntax and vocabulary in conversation. The inconsistency in terms of changes with age within and between diagnostic groups across contexts suggest that there is a need to assess structured expressive language under a broader range of speaking tasks and contexts, both from a research and a clinical perspective. In addition, our results reinforce the importance of considering the role of general NV cognition when it comes to understanding expressive language development of adolescents with DS or FXS. Finally, the observed associations between family-related factors and the trajectory of expressive language suggests that interventions aiming to improve expressive language development of individuals with DS or FXS should acknowledge or address the quality of the mother–youth relationship. Further research is needed to determine how best to capitalize on these results of this study in terms of developing treatments centered in the mother–youth relationship while considering the constellation of material, intellectual, and social resources and experiences of each family. Working on this relationship while considering independent resources, may provide added benefit to youth with DS or FXS receiving speech and language interventions.

## Data Availability

The datasets used and/or analyzed during the current study are available from the corresponding author on a reasonable request.

## Abbreviations

ASD: Autism spectrum disorder
CA: Chronological age
DS: Down syndrome
FMR1: Fragile X mental retardation 1 gene
FMRP: Fragile X mental retardation 1 protein
FXS: Fragile X syndrome
GSI: General severity index
ID: Intellectual disability
IQ: Intellectual quotient
M: Mean
MA: Mental age
PAI: Positive Affect Index
SCL-90-R: The Symptom Checklist-90 Revised
SD: Standard deviation
TD: Typically developing
